# MtOrt: an empirical mitochondrial amino acid substitution model for evolutionary studies of Orthoptera insects

**DOI:** 10.1186/s12862-020-01623-6

**Published:** 2020-05-19

**Authors:** Huihui Chang, Yimeng Nie, Nan Zhang, Xue Zhang, Huimin Sun, Ying Mao, Zhongying Qiu, Yuan Huang

**Affiliations:** 1 College of Life Sciences, Shaanxi Normal University, No. 620, West Chang’an Avenue, Xi’an, 710119 Shaanxi China; 2 School of Basic Medical Sciences & Shaanxi Key Laboratory of Brain Disorders, Xi’an Medical University, Xi’an, 710021 China

**Keywords:** Amino acid substitution model, Mitochondrial genome, Orthoptera, Phylogeny

## Abstract

**Background:**

Amino acid substitution models play an important role in inferring phylogenies from proteins. Although different amino acid substitution models have been proposed, only a few were estimated from mitochondrial protein sequences for specific taxa such as the mtArt model for Arthropoda. The increasing of mitochondrial genome data from broad Orthoptera taxa provides an opportunity to estimate the Orthoptera-specific mitochondrial amino acid empirical model.

**Results:**

We sequenced complete mitochondrial genomes of 54 Orthoptera species, and estimated an amino acid substitution model (named mtOrt) by maximum likelihood method based on the 283 complete mitochondrial genomes available currently. The results indicated that there are obvious differences between mtOrt and the existing models, and the new model can better fit the Orthoptera mitochondrial protein datasets. Moreover, topologies of trees constructed using mtOrt and existing models are frequently different. MtOrt does indeed have an impact on likelihood improvement as well as tree topologies. The comparisons between the topologies of trees constructed using mtOrt and existing models show that the new model outperforms the existing models in inferring phylogenies from Orthoptera mitochondrial protein data.

**Conclusions:**

The new mitochondrial amino acid substitution model of Orthoptera shows obvious differences from the existing models, and outperforms the existing models in inferring phylogenies from Orthoptera mitochondrial protein sequences.

## Background

Amino acid substitution models (models for short) play an important role in many aspects of protein analyses such as measuring the genetic distance, aligning protein sequences or inferring phylogenies [1, 2]. The first molecular sequences to be used for phylogenetic inference were proteins [3].

The standard amino acid substitution model consists of two components: a 20 × 20 instantaneous substitution rate matrix and a vector of 20 amino acid frequencies. There are two main approaches to estimate amino acid substitution models, the parsimony approach and the maximum likelihood approach [2]. The first parsimony method was proposed by Dayhoff et al. [4] to estimate the PAM model (Dayhoff model). Then, on the basis of Dayhoff model, other alternative models based on parsimony method, such as JTT [5], BLOSUM62 [6], VT [7], were proposed successively. The parsimony methods are fast, but they are limited to only pairwise protein alignments and closely related amino acid sequences. The maximum likelihood (ML) method was proposed by Adachi and Hasegawa [8] to estimate the mtREV model with fully utilizing the information contained in multiple protein alignments and the corresponding phylogenetic trees, which must be estimated from the data [4, 5, 9–11].

As more protein sequences accumulated, a number of models have been determined for general interest proteins, such as WAG [10], LG [11]. Although these general models have been calculated from broad taxonomic groups, it has been shown that models specific to certain protein groups (e.g. mitochondrial) or life domains (e.g. viruses) differ significantly from general models, and thus perform better when applied to the data to which they are dedicated [12]. A number of specific amino acid substitution models have been introduced, e.g. cpREV (chloroplast proteins model) [13], rtREV (retrovirus-specific model) [14], HIV-specific models [15], FLU (influenza proteins model) [2] and DEN (dengue viruses model) [16].

Mitochondrial genome (mitogenome) encodes proteins have been used extensively as molecular markers for the inference of phylogeny [17–21]. Few groups have estimated empirical models from mitochondrial proteins (mt models). The first mt model is mtREV [8] from 20 vertebrate mitogenomes. Following the observation that differences exist between taxonomic groups, mt models specific to a given lineage have also been developed, such as mtMam [22, 23], MtArt [24], mtPan [25]/mtPan^2013^ [26], MtZOA [27], mtFish [23], and mtMet, mtVer, mtInv, mtPro and mtDeu [28].

A problem with existing empirical models is that they are based on the comparison of restricted datasets. The mt models might over-fit to training data due to a large number of free parameters of the amino acid substitution model (precisely 208 free parameters) and not fit for other lineages [2, 9, 27, 28]. Orthoptera is the most diverse order of polyneopteran insects, and the number of Orthoptera mitogenome sequences increased rapidly. This provides the opportunity to estimate amino acid substitution model that best fits the Orthoptera mt protein sequences. Here, 54 new mitochondrial genome sequences were determined, and a new mitochondrial amino acid substitution model for Orthoptera was estimated by maximum likelihood method based on 283 Orthoptera mitochondrial genomes. We then compared the differences between the new model and the existing model, and the fitting of the mtOrt to the Orthoptera datasets. Finally, we used mtOrt and existing models to explore the phylogenetic relationships of the major Orthoptera lineages and evaluate the performance of the new model in phylogenetic analyses.

## Results

### Fifty-four new mitogenomes

The 54 newly determined mitogenome sequences are available from GenBank (Additional file 1: Table S1), including 53 Caelifera species and 1 Ensifera species. The size of the complete mitogenome sequences of 54 species ranges from 14,957 bp to 16,437 bp. The mitogenomes of all species contain a conserved set of 37 genes, including 13 PCGs, large and small rRNAs (*rrnL* and *rrnS*), 22 transfer RNAs (tRNAs) and a large non-coding region called the A + T-rich region or control region. Among all the Caelifera mitogenomes sequenced in this study, there is an arrangement order translocation of *trnK* and *trnD* (KD rearrangement) was found in 52 species except *Yunnantettix bannaensis* (Caelifera: Tetrigidae). The KD rearrangement was also not found in *Ruidocollaris convexipennis* (Ensifera: Tettigoniidae), but *trnY*-CR-*cox1* rearrangement occurred.

### The new model and its fit to training dataset

The amino acid exchangeability rates and amino acid frequencies of the new model are shown in Table 1. The exchangeability rates between different amino acids varies widely. The highest exchangeability rate (between Asp (aspartic acid) and Glu (glutamic acid), 10.55) is 196,311 times higher than the lowest (between Arg (arginine) and Phe (phenylalanine), 0.00005). The amino acid frequencies of different amino acids are also different, from 0.01 (Arg) to 0.16 (leucine, Leu).

**Table 1:** The mtOrt model

		Ala	Arg	Asn	Asp	Cys	Gln	Glu	Gly	His	Ile	Leu	Lys	Met	Phe	Pro	Ser	Thr	Trp	Tyr	Val
Exchangeability rates	Ala																				
	Arg	0.04																			
	Asn	0.03	0.22																		
	Asp	0.14	0.09	6.01																	
	Cys	0.64	1.63	0.57	0.24																
	Gln	0.09	3.01	1.44	0.30	0.25															
	Glu	0.20	0.03	1.64	10.55	0.25	2.50														
	Gly	1.05	0.21	0.69	1.13	1.25	0.08	1.24													
	His	0.07	1.43	2.55	0.46	0.24	5.73	0.12	0.08												
	Ile	0.08	0.04	0.44	0.05	0.28	0.05	0.06	0.04	0.10											
	Leu	0.06	0.07	0.07	0.02	0.28	0.23	0.05	0.03	0.14	1.59										
	Lys	0.00	1.43	3.95	0.13	0.00	3.98	3.33	0.13	0.25	0.08	0.07									
	Met	0.41	0.02	0.43	0.05	0.27	0.25	0.29	0.17	0.14	2.95	3.98	0.80								
	Phe	0.06	0.00	0.12	0.02	1.50	0.06	0.07	0.09	0.17	1.00	2.16	0.03	0.63							
	Pro	0.52	0.42	0.27	0.06	0.00	0.95	0.11	0.01	0.73	0.07	0.29	0.43	0.07	0.08						
	Ser	3.26	0.25	2.51	0.49	4.04	0.45	0.57	2.47	0.20	0.18	0.45	3.04	0.75	0.62	1.51					
	Thr	4.04	0.09	1.77	0.07	0.34	0.15	0.24	0.03	0.24	2.14	0.27	0.71	3.35	0.09	1.11	3.91				
	Trp	0.03	1.21	0.10	0.19	2.09	0.01	0.18	0.25	0.02	0.07	0.42	0.32	0.21	0.52	0.06	0.34	0.02			
	Tyr	0.02	0.22	1.44	0.41	3.73	0.75	0.27	0.09	4.15	0.17	0.21	0.39	0.30	3.90	0.22	0.52	0.13	0.78		
	Va	2.31	0.06	0.09	0.13	1.69	0.01	0.32	0.54	0.00	9.41	0.84	0.05	2.49	0.66	0.08	0.38	1.51	0.14	0.16	
Amino acid frequencies		0.04	0.01	0.06	0.02	0.01	0.02	0.02	0.04	0.01	0.11	0.16	0.03	0.09	0.09	0.03	0.10	0.06	0.02	0.05	0.05

We evaluated the fit of the new model on the training dataset. Table 2 shows significant likelihood improvements of the new models (*Q’*) over the initial model during the model training process. The first iteration contributed about 98% of the total likelihood improvement. The optimization process of the new model was terminated after the third iteration, as the gain from the third iteration was insignificant. It is obvious that likelihood and AIC improvements of the final model (*Q’* = mtOrt) over the initial model (mtInv) are significant (i.e., 1943.112 and 3470.224, respectively). Compared with the initial model (*Q*), the new model (mtOrt) fit the training dataset better, which is confirmed by the likelihood improvement and better AIC score of the new model [29]. The score guarantee that the likelihood gain of the new model comes from their genuine fit and overwhelm the penalty of free parameters [9, 28].

**Table 2:** Log-likelihood of the target function on training dataset

mtInv (initial model)	− 438,493.411
First iteration	− 436,598.238
Second iteration	−436,551.677
Third iteration (final model)	−436,550.299
AIC improvement	3470.2240000001
AIC/site	0.804

Note: AIC/site are the AIC improvement per site of the final model in comparison to the initial model mtLnv, respectively

### Model evaluation

#### The robustness of new model

The mtOrt model was estimated from the training dataset containing 89.4% of the Orthoptera mt protein sequences. To examine the robustness of the mtOrt model, we estimated additional models from three other datasets, namely mtOrt_O, mtOrt_C and mtOrt_E (Additional file 2: MtOrt_4.nexus.txt). MtOrt_O estimated from the dataset consisting of all Orthoptera mt protein sequences (283 species). MtOrt_E estimated from the dataset containing all Ensifera mt protein sequences (91 species). MtOrt_C estimated from the dataset containing all Caelifera mt protein sequences (192 species). The correlation of frequency vectors between mtOrt and mtOrt_O is equal to 1 and the other are close to 1. The correlations of exchangeability matrices between these four models (Table 3) are significantly higher than that between mtOrt and existing models (Table 4), especially the correlation between mtOrt and mtOrt_O is almost 1. The comparison of frequency vectors of the four models estimated by different datasets revealed that there was no significant difference in the amino acid frequencies between all models, and the *p*-value range is from 0.437 (MtOrt_E - MtOrt_C) to 0.973 (MtOrt - MtOrt_O) (*p* > 0.05). The comparison of exchangeability matrices of the four models also showed that there was no significant difference in the amino acid exchangeability rates between all models, and the p-value range is from 0.999998 (MtOrt - MtOrt_E and MtOrt_E - MtOrt_C) to 1 (MtOrt - MtOrt_C) (p > 0.05). These results further indicate that mtOrt model fits the orthopteran mt protein dataset better than the existing models and is a robust model with parameters stability.

**Table 3:** The correlations between mtOrt, mtOrt_O, mtOrt_C and mtOrt_E. MtOrt_O models

	mtOrt	mtOrt_O	mtOrt_C	mtOrt_E
mtOrt		1.000**	0.998**	0.998**
mtOrt_O	0.999**		0.998**	0.998**
mtOrt_C	0.990**	0.990**		0.992**
mtOrt_E	0.986**	0.985**	0.954**	

Note: The values in the top triangle represent the correlations between frequency vectors, while values in the low triangle are the correlations between exchangeability matrices. The greater the absolute value of the Pearson correlation coefficient, the higher the correlation. **: *p* < 0.01, extremely significant correlation

**Table 4:** The Pearson’s correlations between 12 models: mtOrt and 11 widely used models

	Dayhoff	JTT	LG	mtArt	mtDeu	mtInv	mtMet	mtPan2013	mtPro	mtZoa	WAG	mtOrt
Dayhoff		0.903**	0.812**	0.397	0.903**	**0.292**	0.365	0.34	0.903**	0.495*	0.93**	0.252
JTT	0.896**		0.961**	0.559*	1.000**	0.444	0.523*	0.517*	1.000**	0.643**	0.975**	0.436
LG	0.854**	0.914**		0.541*	0.961**	0.457*	0.527*	0.538*	0.961**	0.619**	0.912**	0.457*
mtArt	0.767**	0.789**	0.866**		0.559*	0.941**	0.965**	0.964**	0.559*	0.981**	0.491*	0.948**
mtDeu	0.895**	1.000**	0.914**	0.789**		0.444	0.523*	0.517*	1.000**	0.643**	0.975**	0.436
mtInv	0.024	−0.015	0.028	0.091	−0.014		0.976**	0.968**	0.444	0.875**	0.363	0.981**
mtMet	−0.025	−0.018	0.056	0.109	−0.017	0.959**		0.982**	0.523*	0.929**	0.439	0.983**
mtPan2013	−0.035	−0.025	**0.002**	0.054	−0.024	0.956**	0.897**		0.517*	0.931**	0.434	0.988**
mtPro	0.896**	1.000**	0.914**	0.789**	1.000**	−0.015	−0.018	−0.025		0.643**	0.975**	0.436
mtZoa	0.821**	0.825**	0.894**	0.982**	0.852**	0.072	0.086	0.037	0.852**		0.578**	0.892**
WAG	0.919**	0.934**	0.961**	0.809**	0.934**	0.003	0.017	−0.016	0.934**	0.850**		0.347
mtOrt	−0.046	−0.046	− 0.011	0.047	− 0.440	0.952**	0.929**	0.927**	−0.046	0.025	−0.024	

Note: The values in the top triangle represent the correlations between frequency vectors, while values in the low triangle are the correlations between exchangeability matrices. The greater the absolute value of the Pearson correlation coefficient, the higher the correlation. *: *p* < 0.05, significant correlation; **: *p* < 0.01, extremely significant correlation

#### Model comparisons

We measured the correlations between mtOrt and other 11 widely used existing models (Table 4). For the exchangeability rate matrices, the lowest correlation among the 12 models is between mtPan^2013^ and LG models, and the highest is among JTT, mtDeu and mtPro models. Compared with the new model, mtInv is the closest model to mtOrt in terms of exchangeability rates and LG has the lowest correlation. For the frequency vectors, the lowest correlation among the 12 models is between Dayhoff and mtInv models, and the highest is among JTT, mtDeu and mtPro models. MtPan^2013^ model is the closest to the amino acid frequency of mtOrt model and Dayhoff has the lowest correlation. MtInv, mtMet and mtPan^2013^ are most highly correlated with mtOrt and have significant correlations, both in exchangeability matrix and frequency vector (*p* < 0.01).

**Fig. 1:**
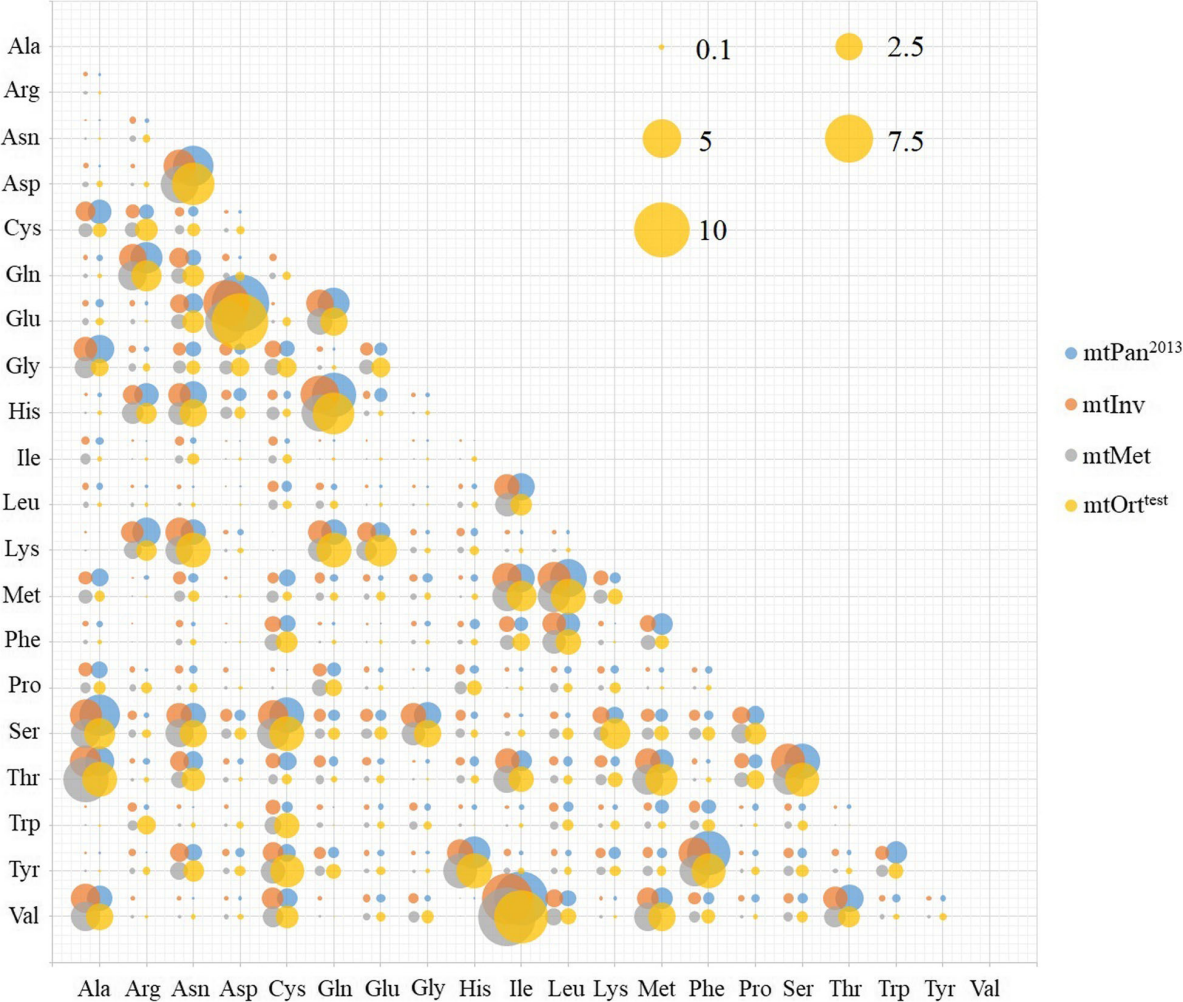
Amino acid exchangeability rates of mtOrt, mtInv, mtPan^2013^ and mtMet models

**Fig. 2:**
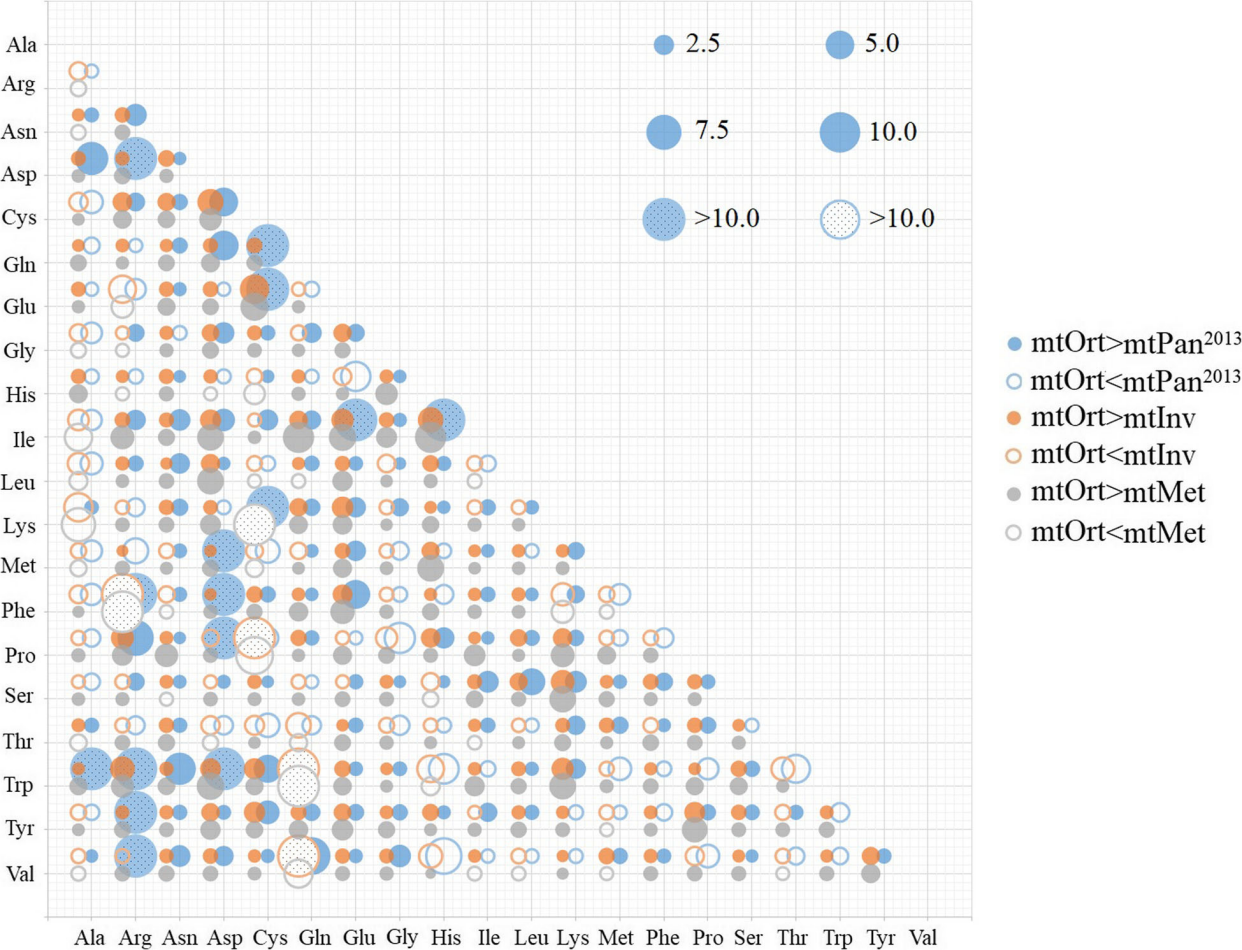
The ratio of exchangeability rates between mtOrt and mtMet/mtPan^2013^/mtInv models. The size of one circle represents the exchangeability rate between mtOrt and other models. The solid (unfilled) circles represent exchangeability rates where mtOrt is bigger (smaller) than the three models. For visualization, the large ratios are trimmed at 10 and marked with dotted circles

Based on the results of correlation analysis, we compare the differences between the new model and the existing models. The amino acid exchangeability rates of mtOrt, mtInv, mtPan^2013^ and mtMet models were plotted in Fig. 1. In mtInv and mtMet models, the exchangeability rates between Val (valine) and His (histidine) are the lowest (0.008 and 0.004), and that between Val and Ile (isoleucine) are the highest (8.543 amd 10.953). The rates between Glu and Asp (asparagine) are the highest in Pan^2013^ (10.819) and mtOrt (10.552), but the lowest rate in Pan^2013^ is between Arg and Asp (0.00000001), while the lowest rate in mtOrt is between Arg and Phe (0.00005). The change of amino acid exchangeability rates between different models is basically the same. However, they differ considerably when we look in their relative differences (Fig. 2). For example, the coefficients on Ala (alanine) row are notably different among models, most of them are mtOrt < mtPan^2013^/mtInv. The 15 out of 190 coefficients in mtOrt are at least 10 times as large as corresponding ones in the mtPan^2013^ model. MtInv and mtMet models have 4 and 3 coefficients that are at least 10 times larger than mtOrt, respectively.

**Fig. 3:**
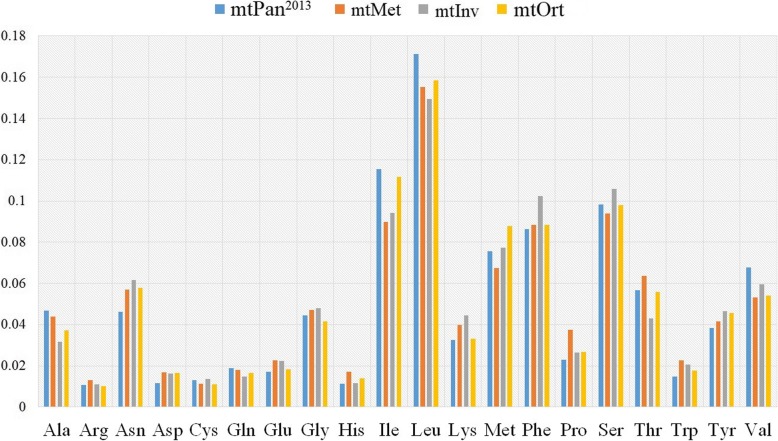
Amino acid frequencies of mtOrt, mtInv, mtPan^2013^ and mtMet models

Amino acid frequencies of the four models, mtOrt, mtInv, mtMet and mtPan^2013^, are nearly identical (Fig. 3, correlation > 0.98), their correlation being much higher than other models (Table 4). We observed some notable differences between frequencies of these models. For instance, the frequency of Met in mtOrt (0.09) is higher than other three models and is 1.3 times than that in mtMet (~ 0.07), while Gly (glycine) frequency is only 0.04 in mtOrt, which is the lowest in all models.

#### Phylogenetic performance

We assessed the performance of the new model and the existing models on building maximum likelihood phylogenies. For each dataset, we optimized parameters of the rate heterogeneity model, including proportion of invariable sites and shape of Gamma distribution with 4 categories, but fixed the exchangeability rates and base frequencies of the models.

**Fig. 4:**
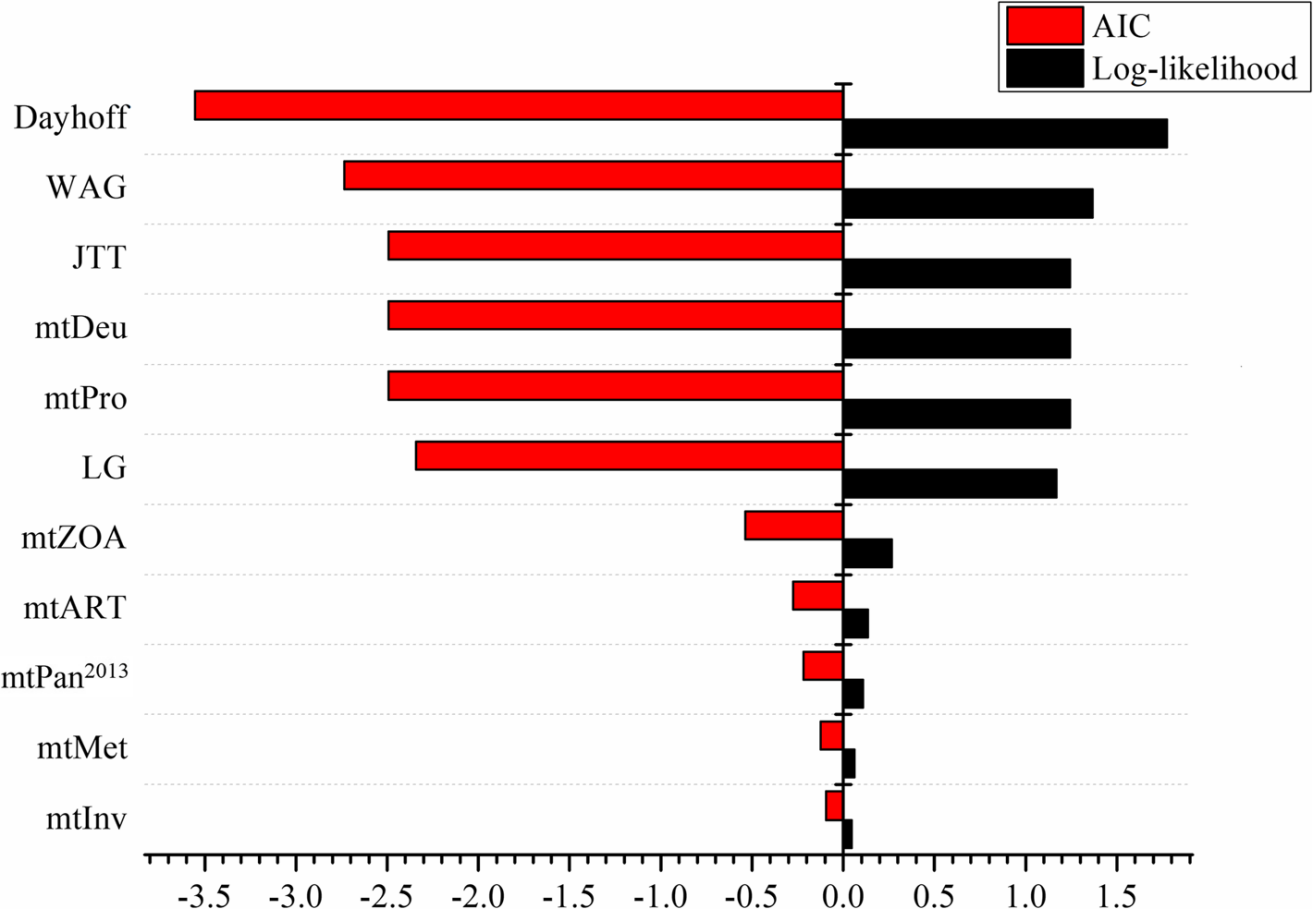
The mean difference of log-likelihood and AIC scores of per site between mtOrt and the existing models on testing datasets

We calculated the mean differences of the log-likelihood and the AIC score of per site (AIC/site) for testing datasets between mtOrt and other 11 models. It is clear that the mean differences of AIC/site between mtOrt and other models are negative, and the differences of log-likelihood are positive, which indicate that mtOrt outperform the existing models for testing datasets, followed by mtInv, mtMet, mtPan^2013^, mtArt, mtZoa (Fig. 4). Furthermore, we compared the performance of new model to LG4X and C60 (site-heterogeneous models) [29]. The results illustrate that the new model outperformed LG4X and C60 models.

The whole dataset, which include 283 Orthoptera mt protein sequences, was divided into sub-datasets with two algorithm, and different k values targeting sub-dataset sizes of 16, 24, 32, 64 and 120 sequences [9]. Using the random splitting algorithm, 43 sub-datasets (RSDs) were obtained and the tree-based splitting algorithm obtained 42 sub-datasets (TSDs). First, we evaluated the best-fit model for 85 sub-datasets by ModelFinder [30]. The results show that the best-fit models for all RSDs are mtOrt. Most of the best-fit models of TSDs are mtOrt, but there are six TSDs where the best-fit models are mtMet, and two of them are obtained by k = 32, four are obtained by k = 16.

**Fig. 5:**
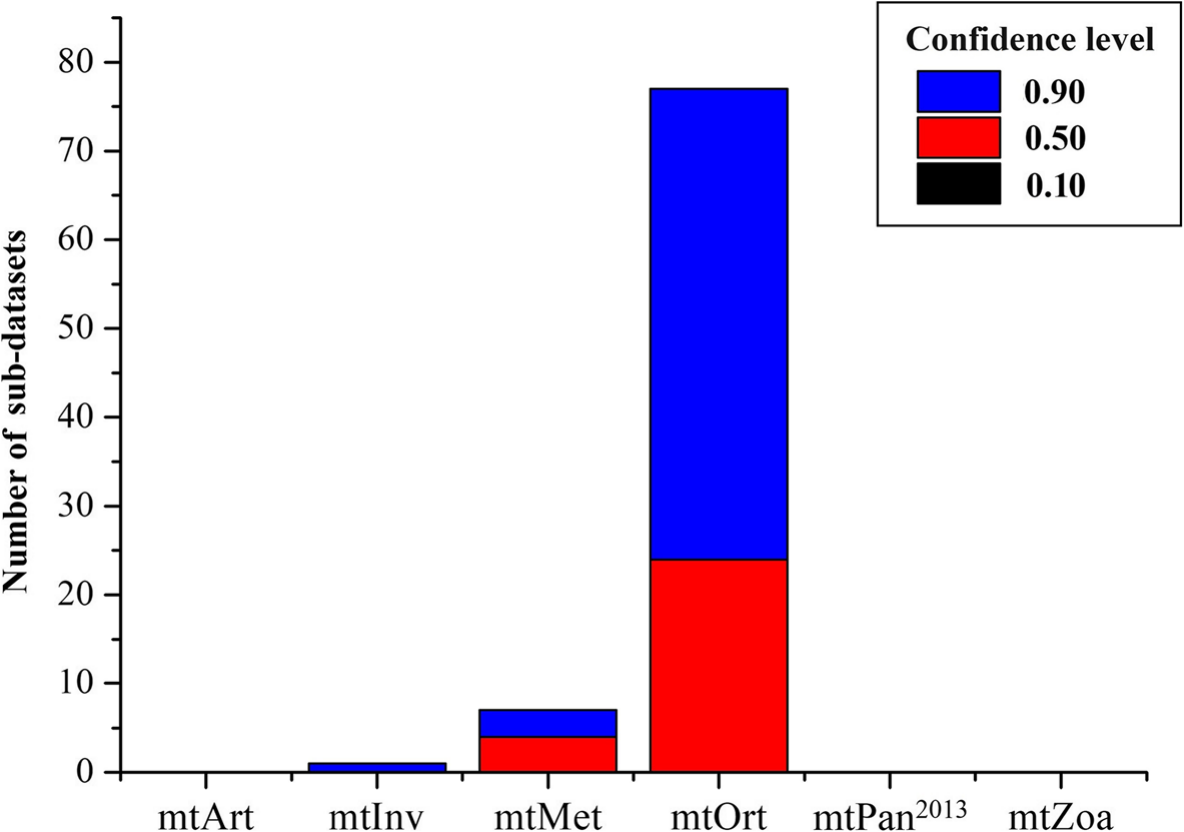
The number and confidence levels of different models that build the optimal topology for each sub-dataset

Next, we evaluated the performance of mtOrt and other five models (mtInv, mtPan^2013^, mtMet, mtArt and mtZoa) by comparing the log-likelihood of trees (each sub-dataset has six trees, involving a total of 510 trees), which were inferred from each sub-dataset by IQ-TREE 1.7 with different models. The performance of the mt models at the individual dataset were estimated by approximately unbiased test (AU test) for phylogenies [29, 31, 32]. The CONSEL program was used to assess the confidence levels of the site log-likelihoods for phylogenies with the different models of each sub-dataset. The results of AU test show that among the 85 sub-datasets, the best log-likelihood of trees of 77 datasets are constructed by mtOrt model, and these 77 sub-datasets (90.6%) only accept the topologies constructed by mtOrt, while significantly rejecting the topologies built by five existing mt models, and 68.8% of them have a confidence level of 0.9 (Fig. 5). The mtMet are the best-fit models for 7 out of 85 sub-datasets, but only significantly better for three datasets at the 0.9 confidence level, while the mtInv only significantly better for one sub-dataset at the 0.9 confidence level, and they are all smaller data sets. The other five existing models were not the best-fit model for any datasets.

**Fig. 6:**
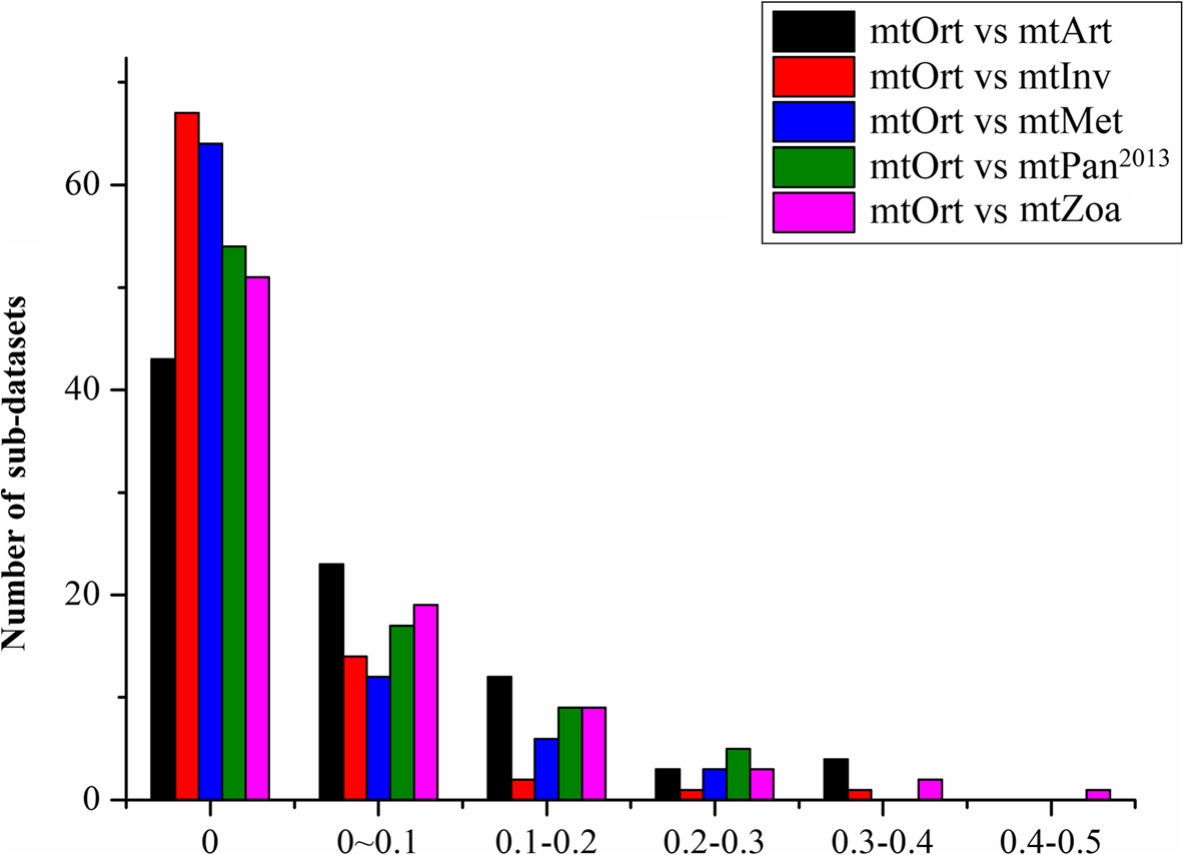
The topological distances between trees inferred using mtOrt and five existing models. The horizontal axis indicates the topological distance between 2 tree topologies, whereas the vertical axis indicates the number of datasets

We investigated the topological quality of phylogenies for each testing datasets and sub-datasets with six mt models (mtOrt, mtInv, mtPan^2013^, mtMet, mtArt and mtZoa) by measuring their topological distances from the best phylogenies. Specifically, we used the Matching Split distance (MS) metric to measure the distance between two phylogenies by TreeCmp 2.0 [33]. Although no difference was detected in the topologies of the testing datasets built by different models, Fig. 6 discloses remarkable topological distances from the phylogenies of sub-datasets with existing models to the new model. For 85 sub-datasets, the phylogenies built by mtInv and mtOrt have the same topologies for 67 sub-datasets, and the phylogenies of 64, 54, 51 and 43 sub-datasets inferred by mtMet, mtPan^2013^, mtZoa and mtArt have the same topologies as that constructed by mtOrt, respectively. The topologies inferred by mtArt are different from that constructed by mtOrt in 49.4% of sub-datasets, and the phylogenies of 40.0%, 36.5, 24.7 and 21.2% sub-datasets inferred by mtZoa, mtPan^2013^, mtMet, and mtInv are different from that constructed by mtOrt, respectively. We also compared the node support values of the trees constructed by different models for testing datasets and sub-datasets. The results showed that the new model did not improve the node support values, and the node support values of mtOrt_trees are not significantly different from those of the existing models (*p* > 0.05).

We used Polyneoptera mitogenomes dataset to test whether the new model would be used in phylogenetic estimation of other closely related taxa. For the trees of Polyneoptera constructed by different models, mtOrt_tree, mtInv_tree, mtMet_tree and mtPan^2013^_tree have the same topology (Additional file 3: Figure S1). Although mtOrt_tree does not have the optimal likelihood and AIC, it has the optimal node support value, and there is no significant difference between different models (p > 0.05). The results indicated that the new model is also applicable in the study of phylogenetic relationship of Polyneoptera.

### Phylogenetic analysis of Orthoptera

The 14 Orthoptera phylogenetic trees (inferred by the new model, 11 existing models and two site-heterogeneous models (LG4X and C10)) show that mtOrt (+R10) resulted in a likelihood advantage over other models (1812.897 log-likelihood advantage over the second-best model, mtInv (+R10)). The AU test supports that mtOrt_tree is optimal (au = 1.000 and *p* < 0.01), and significantly rejects the topologies of other trees (the au values of the other 13 trees are less than 0.01, and the *p* values are less than 0.01). By comparing the topologies, the abnormal result of the clustering of grylloid (include Grylloidea and Gryllotalpoidea of Ensifera) and Caelifera is found in all the nine trees (mtArt_tree, mtZoa_tree, LG_tree, mtPro_tree, JTT_tree, mtDeu_tree, WAG_tree and Dayhoff_tree). The topology constructed by site-heterogeneous models (LG4X and C10) also performs poorly.

**Fig. 7:**
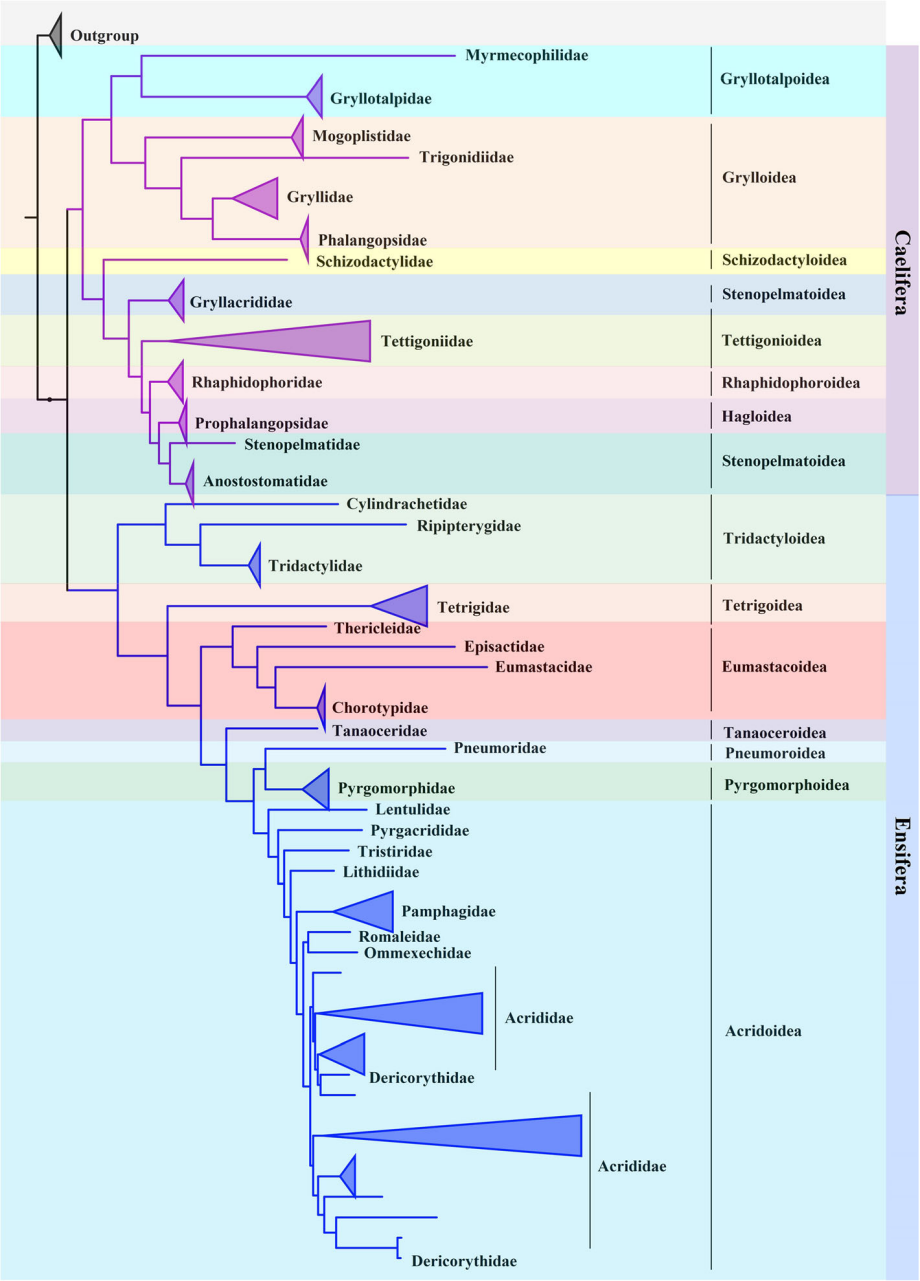
The phylogenic relationships among the higher taxa of Orthoptera

The comparisons between mtOrt_tree and mtMet_tree, mtInv_tree and mtPan^2013^_tree shows that the relationships between higher-level taxa are identical and very stable (Fig. 7). The MS metric was used to measure the distance between four phylogenies. The result shows that the four topologies are very similar to each other, The MS distances range from 0.0025 (Pan^2013^_tree vs mtMet_tree) to 0.0201 (mtMet_tree vs mtInv_tree). The most similar to mtOrt_tree is mtInv_tree (0.0062), followed by mtMet_tree (0.0161) and mtPan^2013^_tree (0.0175).

Overall, Orthoptera is divided into two large branches: Ensifera and Caelifera (Fig. 7). Within the Ensifera, the relationships among the seven superfamilies were ((((Tettigonioidea + ((Stenopelmatoidea + Hagloidea) + Rhaphidophoroidea)) + Stenopelmatoidea) + Schizodactyloidea) + (Grylloidea + Gryllotalpoidea)). Within the Caelifera, the relationships among the seven superfamilies were ((((((Pyrgomorphoidea + Pneumoroidea) + Acridoidea) + Tanaoceroidea) + Eumastacoidea) + Tetrigoidea) + Tridactyloidea). By comparing the topological structure of four trees (mtOrt_tree, mtMet_tree, mtInv_tree and mtPan^2013^_tree), we found eight differences (two in the branch of Ensifera and six in the branch of Caelifera), and all of them appeared in the lower classification level (Additional file 4: Figure S2).

## Discussion

### Differences between different models

Through the comparison of different models, the low correlations of the 12 models are found, which confirm high variation among the models. We observed remarkably low correlations between mt models and general models (e.g., the 0.002 correlation score between mtPan^2013^ and LG) (Table 4). Thus, general models are not an appropriate choice in inferring phylogenies from mt protein data [28]. The low pairwise correlations of exchangeability rate matrices (or frequency vectors) between mtOrt and other models mean that mtOrt is significantly different from existing models. As expected, mtInv is the closest model to mtOrt in terms of exchangeability rates, with a 0.952 correlation score, as both were trained from the invertebrate data. Interestingly, mtOrt is closer to mtInv than mtArt, which indicate diverse evolutionary processes among different lineages.

For different models, the change trend of amino acid replacement rates between different amino acids and amino acid frequency is basically the same [2, 16, 26, 28]. In general, most values distributed in a similar trend due to biological constraints [2, 24, 28], such as the high exchange rate between Lys (lysine) and Arg (two positively charged, polar amino acids), aspartic acid and glutamic acid (two negatively charged, polar amino acid) or the low exchange rate between Lys and Cys (cysteine) (a neutral, nonpolar amino acid). Ile is frequently substituted by Val, Met (methionine), Leu, Thr (threonine) and Phe (hydrophobic amino acids), while other amino substitution rarely happen as their corresponding rates are relatively small (Fig. 1) [2, 34]. However, we still find some obvious differences of exchangeability rates and amino acid frequencies between mtOrt and mtInv, mtMet and mtPan^2013^ models (Fig. 2 and Fig. 3), which indicate that mtOrt represents the exchangeability rates and amino acid frequencies of Orthoptera mt proteins more accurately than other models.

### Phylogenetic improvement of the new model

#### Likelihood improvement on different datasets

For the testing datasets, compared with the existing model, the likelihood improvement indicates that mtOrt model can not only fit the training dataset participating in the construction of the new model, but also better fit the testing datasets that are not involved in building the new model (Fig. 4).

For the 85 sub-datasets, from the results of ModelFinder, the new model also demonstrates a better fit for almost all sub-datasets in comparison with the existing models, the proportion of mtOrt reaches 93% in all sub-datasets. Although the best-fit models of six TSDs are the existing model (mtMet), all the species in these relatively small sub-datasets are part of Tettigoniidae, which indicates that the evolutionary patterns of different lineages of Orthoptera are also different. The AU test and confidence level results of the log-likelihoods for phylogenies constructed by different models of each sub-dataset are congruent with that of model selection by ModelFinder, which confirms the significantly superiority of the new model with high confidence levels in inferring phylogenies for all sub-datasets than existing models (Fig. 5).

**Fig. 8:**
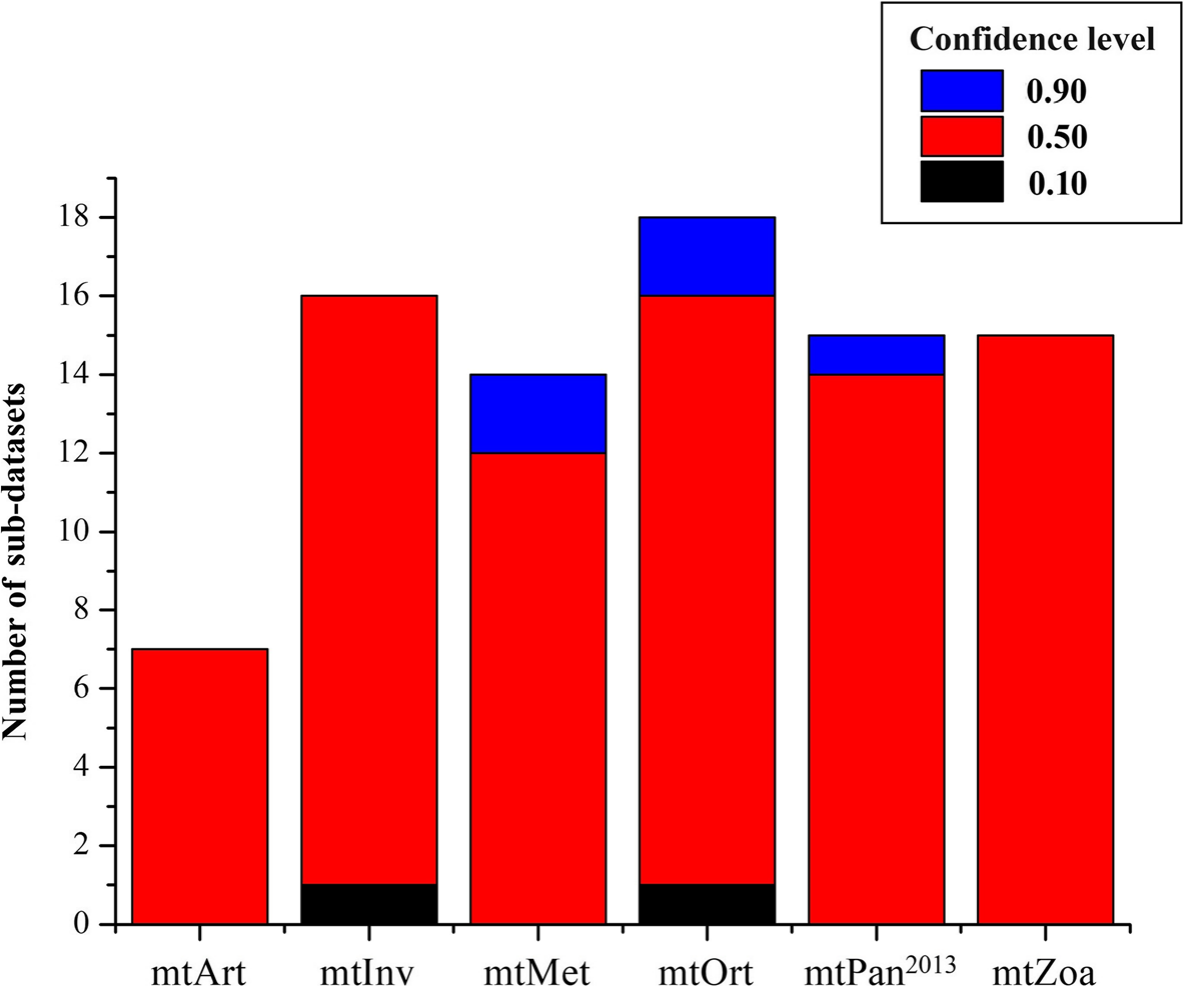
The number and confidence levels of different models optimized by mtOrt (+R5) that build the optimal topology for each sub-dataset

In order to verify that the likelihood improvement of the new model is derived from the parameters of mtOrt model rather than other factors, the AU test was also used to examine the parameters of the different models that have been re-optimized by the best-fit models. We used ModelFinder to select the most suitable model from 12 models (mtOrt and 11existing models) without any model parameter optimization for the testing dataset of 30 species. The result shows that the best-fit model is mtOrt+R5, so we assume that mtOrt+R5 is the optimal model for all sub-datasets and use that model to build the ML tree for all sub-datasets. Then, IQ-TREE 1.7 was used to recalculate the log-likelihood of the trees, which were built from the different models in the previous analysis for each sub-dataset, based on the estimated parameters done for the ML tree. That is to say, we use mtOrt (+R5) to fix the topology of the trees and use the parameters of mtOrt (+R5) to re-optimize other parameters (branch lengths, parameters of rate heterogeneity model) of the trees constructed by other models [28, 29]. Then we used the CONSEL program for assessing their confidence levels. The results reveal that the number of different models that are superior to the other five models for 85 sub-datasets are 18 (mtOrt), 16 (mtInv), 15 (mtPan^2013^), 15 (mtZoa), 14 (mtMet), 7 (mtArt), and most of them have lower confidence levels (Fig. 8). It is reveals that the trees built with the new models are still better than that with the existing models in term of likelihood, but the proportion is reduced (from 90.6 to 21.2%) and with lower confidence. Although the proportion of existing models has increased (from 9.4 to 78.8%), they have lower confidence levels. In the AU test, it is not found that any sub-dataset only accept the topology constructed by mtOrt, while rejecting the topology built by five existing mt models. The increase of the proportion shows that the parameters of the existing models re-optimized by mtOrt (+R5) are improved, and they fit better with the corresponding datasets, which further indicates that the parameters of the new model are better than the existing models. The significant drop of confidence levels of all models reveals that a large proportion of likelihood gain is due to the new models other than tree topologies [28, 32].

We further investigated the performance of the new model for individual mt protein dataset. In the 13 protein datasets, most of the best-fit models are mtOrt, followed by mtInv, mtMet and mtPan^2013^ and the worst performer was Dayhoff model. Only the optimal models for *ND4* and *ND5* are mtInv, followed by mtOrt, and there is little difference between the values of log-likelihood, AIC and BIC.

#### Topology improvement on different datasets

We use MS distance to estimate the topology differences between the new model and the existing models for all datasets. One of the advantages of the MS distance is its natural character; i.e., the definition is based on splits, similarly to the Robinson-Foulds (RF) metric. On the other hand, the MS distance is more sensitive than RF and is resistant to displacement of a small number of leaves [35]. The normalized MS distances divided by pre-computed empirical average values for random trees (generated according to Yule and uniform models) can help in an interpretation of the similarity level of analyzed trees in chosen metric [33]. Although the testing datasets and more than half of the sub-datasets (50.6% (mtArt) ~ 78.8% (mtInv)) have the same topologies inferred using existing models as the mtOrt tree, the results also show that topologies of other sub-datasets inferred using mtOrt are different from those inferred using other models. For example, the MS distance between mtOrt trees and mtPan^2013^ is 0 ~ 0.1 (0.1 ~ 0.2) for about 20% (10.6%) of sub-datasets (Fig. 6). The results reconfirm the advantage of the new model in improving the topology inference of phylogeny and the essential role of model selections in inferring phylogenies as a poor model selection would lead to low quality phylogenies [28].

### Phylogenetic relationships of Orthoptera lineages

The phylogeny of Orthoptera has been contentious over the years and numerous hypotheses have been proposed based on different character systems [18, 28, 36]. The AU test confirmed that the phylogeny of Orthoptera inferred by mtOrt model is the best among the 14 trees. The results of topology comparison of 14 trees show that the occurrence of abnormal branches in the phylogenies constructed by 11 existing models (mtArt, mtZoa, LG, mtPro, JTT, mtDeu, WAG, Dayhoff, LG4X and C10) further reflect the importance of choosing appropriate models to construct correct evolutionary relationships. Only four models (mtOrt, mtMet, mtInv and mtPan^2013^) accurately inferred the phylogenetic relationship at the suborder level, and the MS distances divided by pre-computed empirical average values for random trees (generated according to Yule) show that the topology of mtOrt model is at a high similarity level with that of the three existing models.

In mtOrt_tree, Orthoptera is divided into two suborders: Ensifera and Caelifera (Fig. 7), and this result is supported by many morphological characteristics and molecular data [17, 18, 37–41]. Ensifera is consist of two clades, grylloid and non-grylloid. Within grylloid clade, Grylloidea and Gryllotalpoidea are sister group. Within non-grylloid clade, the basal group is Schizodactyloidea. The monophyly of Stenopelmatoidea and Schizodactyloidea (only one mitochondrial genome of one species is available in GenBank database) is not supported, and the other five superfamilies are monophyletic [17, 18]. The relationships between these families are agree with previous studies [17, 41, 42]. Caelifera is also divided into two groups. Tridactyloidea formed the basal clade, as a sister group of all the other caeliferan superfamilies [17, 41, 43]. The monophyly of Pneumoroidea and Tanaoceroidea could not be tested, the other five superfamilies are monophyletic. Among the 20 families of Caelifera examined, only Pamphagidae, Pyrgomorphidae, Chorotypidae, Tetrigidae and Tridactylidae are supported as monophyletic. Due to the involvement of two newly determined Dericorythidae species (*Conophymacris viridis* and *Dericorys annulata*), the monophyly of Acrididae is not supported (Additional file 4: Figure S2), which is inconsistent with previous studies [17, 41, 43–45], in which did not sampled Dericorythidae species. Dericorythidae was once treated as a subfamily within the Acrididae, but Eades (2000) elevated it to the family level on the basis of the presence of a deep groove in the endophallic sclerite and the presence of a pseudoarch in the phallic structures, both of which make members of this family distinct from the other species of the Acrididae [45, 46]. The topological inconsistencies of the four trees only show up in a small branch of Acrididae at the subfamily level. The main reason is that the relationship between Catantopinae and three other subfamilies (Calliptaminae, Cyrtacanthacrdinae and Eyreproclonemidinae) is controversial (Additional file 5: Figure S3). Members of the Catantopinae are highly diverse in terms of morphology and often assumed being a monophyletic taxon based on morphological traits [47]. Molecular results appear to confirm earlier suspicions that the subfamily is not monophyletic. *Stenocatantops* and *Xenocatantops* form a sister group, which was also confirmed by the other studies [48–51]. The remaining inconsistencies between mtOrt_tree and mtMet_tree, mtInv_tree and mtPan^2013^_tree are all concentrated on inter-generic and intra-generic relationships (Additional file 4: Figure S2).

## Conclusions

In this work, 54 mitochondrial genomes have been determined. Based on the mt proteins data from newly determined and existing Orthoptera mitogenomes, we constructed the mtOrt model that has been specifically modeling the evolution properties of Orthoptera mt proteins. Analyses revealed significant differences between mtOrt and existing models in both amino acid frequencies and exchangeability rates. Moreover, the new model is better than existing models in fitting the Orthoptera mt proteins data and inferring the phylogenetic relationship. Multiple phylogenetic analyses show that mtOrt is robust, and better characterizes the evolutionary patterns of Orthoptera mt proteins than existing models. The phylogeny of 283 Orthoptera species inferred from mt proteins with the new model is better than existing models and shows that the relationships between higher-level relationships are very stable and strong support for the phylogeny-based natural classification scheme that proposed by Song et al. (2015). We suggest that mtOrt should be used for the mt proteins analysis of Orthoptera datasets.

## Methods

### Sample collection and DNA extraction

The information on the samples and sequencing technology used in the present study was shown in Additional file 1: Table S1. The samples were preserved in 100% ethanol and stored in a − 20 °C freezer at the Institute of Zoology of Shaanxi Normal University. Total genomic DNA was extracted from the muscle tissue of every individual specimen by a DNeasy Blood and Tissue Kit ((50)-QIAGEN 69504), and then stored at − 20 °C.

### DNA sequencing, annotations and analyses

An Illumina HiSeq 2500 system was used to sequence the DNA of the 54 orthopteran insects (Additional file 1: Table S1) with a 150-bp read length. DNA library construction and sequencing were conducted by the Biomarker Company. Mira 4.0.2 and MITObim 1.7 [52, 53] were used with default parameters to assemble the mitogenomes. Transfer RNAs were identified by MITOS2 (http://mitos.bioinf.uni-leipzig.de/index.py) [54]. The other genes were determined in Geneious Prime [55] (available from http://www.geneious.com) by comparison with other related and reference mitogenomes, and then checked manually.

### Datasets

A total of 283 Orthoptera mitochondrial genomes, included 54 newly determined and 229 published sequences from the NCBI (National Center for Biotechnology Information) (Additional file 6: Table S2). To estimate a substitution model, the 283 mitochondrial genomes are divided into training and testing datasets containing 253 and 30 of sequences, respectively. We used Geneious Prime to extract gene sequences from mitochondrial genomes and translated each protein-coding gene into an amino acid sequence in MEGAX with invertebrate mtDNA genetic code [56]. Amino acid sequences were aligned using MUSCLE program [57], and the alignments of individual genes were concatenated using SequenceMatrix v.1.7.8 [58]. The training dataset was used to estimate new mt model.

### Model estimation

**Fig. 9:**
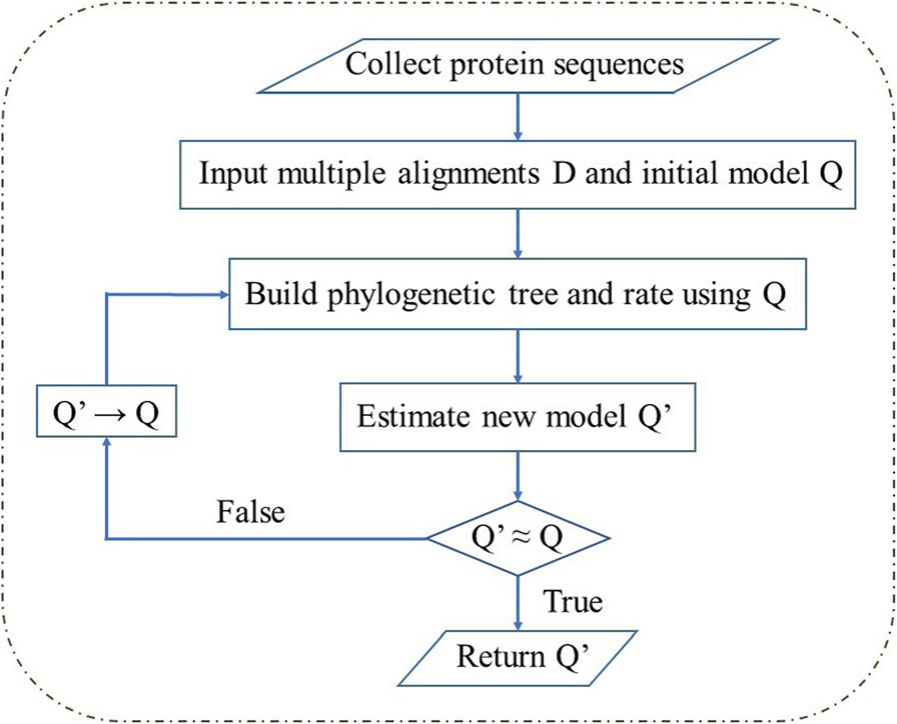
The maximum likelihood-based process to estimate an amino acid substitution model for protein sequences

FastMG [9] was used to estimate the new mt model. We assumed that the standard model for the amino acid substitution process over the tree is a Markov process with time-homogeneous, time-continuous, and time-reversible properties and references therein [19, 28]. The standard model is represented by a 20 × 20 rate matrix *Q* = {*q*_*xy*_} [22], where *q*_*xy*_ (*x* ≠ *y*) is the number of substitution from amino acid *x* to amino acid *y* per time unit. The diagonal elements *q*_*xx*_ are assigned such that the sum of each row equals zero. The matrix *Q* can be decomposed into symmetric exchangeability rate matrix *R* = {*r*_*xy*_} and amino acid frequency vector *Π* = {*π*_*x*_} such that *q*_*xy*_ = *r*_*xy*_*π*_*y*_ and q_*xx*_ = −Σ_*y ≠ x*_*q*_*xy*_. The frequency vector *Π* has 19 free parameters and can be directly approximated from the data. However, the rate matrix *Q* has 190 free parameters and much more difficult to be estimated from the data [10, 59]. In this study, we applied the maximum likelihood method to estimate *Q*. The training dataset was divided into sub-datasets of at most 16 sequences using the tree-based splitting algorithm. Previous studies revealed that the FastMG procedure was an order of magnitude faster than without splitting [9]. The FastMG algorithm starts from an initial model (*Q*) and iteratively optimizes the model until the likelihood improvement is insignificant. The procedure first builds phylogenetic trees and rates using *Q* and maximum likelihood tree construction programs such as PhyML, and then estimates a new exchangeability matrix *Q’* using the approach described by Le and Gascuel [11] and the XRate software [60]. Compare *Q’* and *Q*, if they are nearly identical, return *Q’* as the optimal model. Otherwise, assign *Q* ← *Q’* and re-estimate phylogenetic trees and rates to start a new iteration. Note that mtInv model was assigned as the initial model. A better model *Q* can be estimated from alignments of *D* using an iterative approach as detailed in the 5-step estimation procedure (see Fig. 9).

### Model analysis

The estimation of new model involves 208 additional free parameters, and its likelihood has to be penalized to obtain a fair comparison. The Akaike information criterion (AIC) gain is equal to the twice the log-likelihood gain, minus 416 (= 2 × 208). The penalty (416) is equally divided between all sites in the input alignments. When the AIC gain is positive (negative) for a given alignment, the new model has a better (worse) fit to this alignment than the starting matrix [9, 12]. So we evaluated the fitting of the new model to the training dataset by comparing the gains of likelihood and AIC scores. The testing dataset of 30 species was divided into three smaller datasets by random split method, and the new model was analyzed by four different testing datasets that do not participate in the construction of the new model.

We used IBM SPSS Statistics 20 to compare the correlation between the new model and the 11 existing models (mtInv, mtMet, mtPro, mtDeu, mtPan^2013^, mtArt, mtZoa, LG, JTT, WAG and Dayhoff). The differences of amino acid frequencies and exchangeability rates between the models were analyzed by comparing the new model with existing models.

We evaluated the performance of the new model in different datasets. IQ-TREE 1.7 [29] was used to build phylogenies and estimate the log-likelihood, AIC, AICc (corrected Akaike information criterion) and BIC (Bayesian information criterion) scores of different models on each dataset. ModelFinder [30] was used to find best-fit model of different datasets. The CONSEL program [31] was used for assessing likelihood and confidence levels of different models. The topology differentiation on different datasets was tested by TreeCmp 2.0 [33].

### Phylogenomic analyses

We applied the different models to explore the phylogenetic relationships of the major Orthoptera lineages by the dataset of mt protein sequences from 283 Orthoptera species and outgroup of 3 non-Orthoptera species (GenBank accession No.: NC_034841, NC_034930 and NC_014695) (Additional file 6: Table S2). The result of model selection for the dataset by ModelFinder [30] shows that the models with better performance are optimized by FreeRate model, so we used IQ-TREE 1.7 [29] to infer the phylogenies with the new model, 11 existing models and two site-heterogeneous models (LG4X and C10) and optimised all the models by +R10. We use the models to name the corresponding phylogenetic tree, such mtOrt_tree, and so on. The topological differences between mtOrt_tree and the other 13 trees were compared by the Phylo.io, a web application [61], and evaluated using the CONSEL program [31]. We used the same method to analyze the mt protein data of 23 Polyneoptera species and 3 non-Polyneoptera species (GenBank accession No.: NC_012645, NC_042163 and NC_023232) from GenBank to explore the applicability of the new model to Polyneoptera data (Additional file 6: Table S2).

## Supplementary information

**Additional file 1: Table S1.** Information on the samples used in the present study.

**Additional file 2.** The mtOrt, mtOrt_O, mtOrt_C and mtOrt_E models.

**Additional file 3: Figure S1.** Phylogenetic tree inferred by mtOrt based on mitochondrial proteins of Polyneoptera species.

**Additional file 4: Figure S2.** Phylogenetic trees inferred by mtOrt based on mitochondrial proteins of 286 species. Coloured ranges represent different families. The inconsistent branches between mtOrt_tree and mtMet_tree, mtInv_tree and mtPan^2013^_tree are represented by different colors (Red: mtOrt_tree-mtMet_tree; Green: mtOrt_tree-mtPan^2013^_tree; Yellow: mtOrt_tree-mtInv_tree; Orange: mtInv_tree and mtPan^2013^_tree are the same but different from mtOrt_tree; Red dotted lines: mtMet_tree, mtInv_tree and mtPan^2013^_tree are the same but different from mtOrt_tree.

**Additional file 5: Figure S3.** The topological inconsistencies of the four trees at subfamily level. That is, the position represented by a red dotted lines as shown in Figure S1.

**Additional file 6: Table S2.** Taxonomic information and GenBank accession numbers for the taxa used in this study.

## Data Availability

The sequence data produced and analysed during the current study were deposited in NCBI GenBank (https://www.ncbi.nlm.nih.gov/genbank/) and are freely available under accession numbers MN046211-MN046220, MN083167-MN083209 and MN484604. Other supporting results are included within the article and its additional files. In the Additional file 2: MtOrt_4.nexus.txt, we provide the exchangeability rates and amino acid frequencies of mtOrt, which can be used as a .nexus file in IQ-TREE.

## Abbreviations

Mt: Mitochondrial
Mitogenome: Mitochondrial genome
NCBI: National Center for Biotechnology Information
RSDs: The sub-datasets obtained with random splitting algorithm
TSDs: The sub-datasets obtained with tree-based splitting algorithm
AU: The approximately unbiased test
