# Supplementary figures and images for: MtOrt: an empirical mitochondrial amino acid substitution model for evolutionary studies of Orthoptera insects
Source: BMC Evol Biol. 2020 May 19;20:57. doi: 10.1186/s12862-020-01623-6 (PMC7236349; doi:10.1186/s12862-020-01623-6)

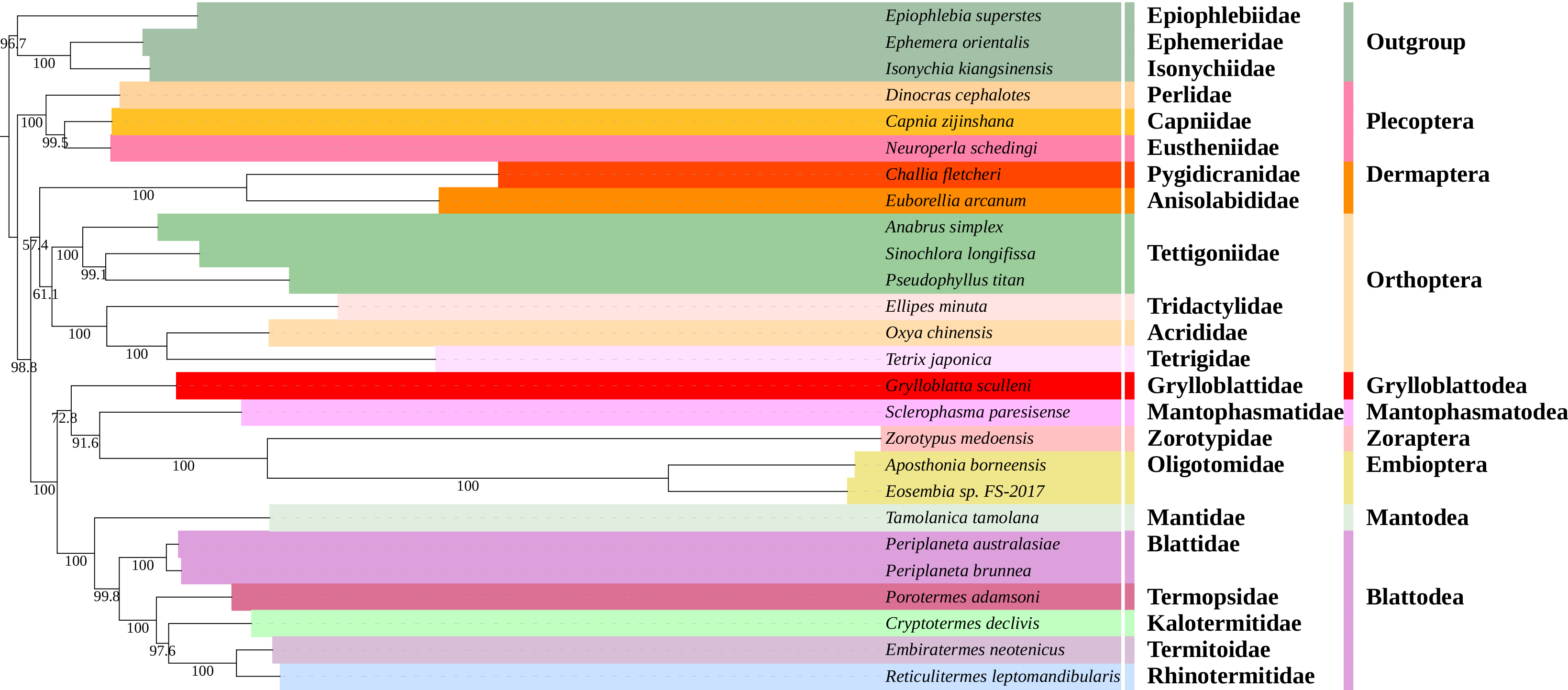

Supplement: Supplementary file 3 — Additional file 3: Figure S1. Phylogenetic tree inferred by mtOrt based on mitochondrial proteins of Polyneoptera species. [file 12862_2020_1623_MOESM3_ESM.jpg]

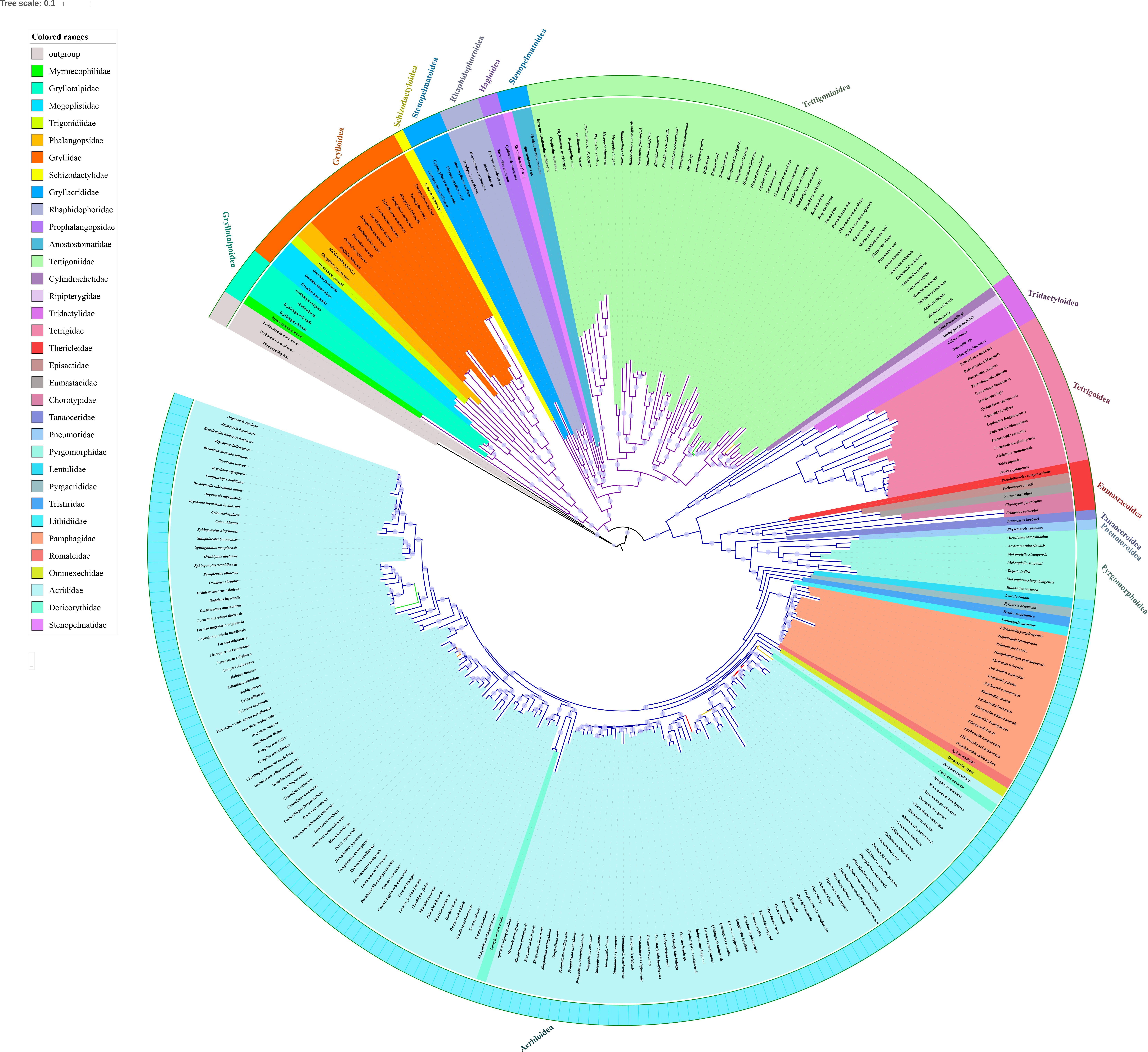

Supplement: Supplementary file 4 — Additional file 4: Figure S2. Phylogenetic trees inferred by mtOrt based on mitochondrial proteins of 286 species. Coloured ranges represent different families. The inconsistent branches between mtOrt_tree and mtMet_tree, mtInv_tree and mtPan2013_tree are represented by different colors (Red: mtOrt_tree-mtMet_tree; Green: mtOrt_tree-mtPan2013_tree; Yellow: mtOrt_tree-mtInv_tree; Orange: mtInv_tree and mtPan2013_tree are the same but different from mtOrt_tree; Red dotted lines: mtMet_tree, mtInv_tree and mtPan2013_tree are the same but different from mtOrt_tree. [file 12862_2020_1623_MOESM4_ESM.jpg]

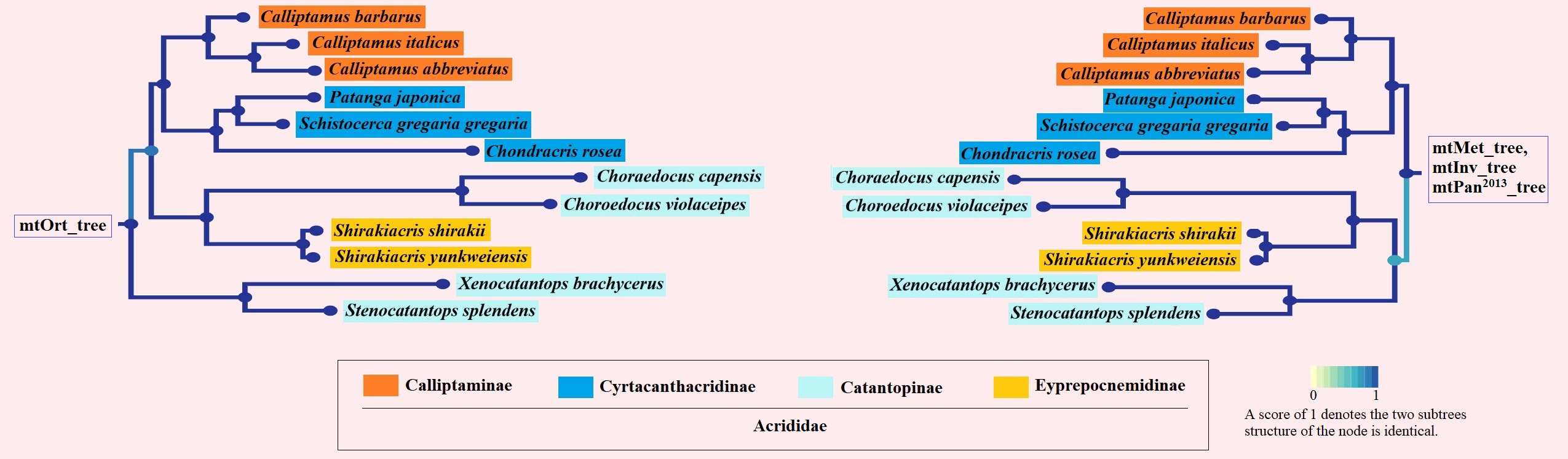

Supplement: Supplementary file 5 — Additional file 5: Figure S3. The topological inconsistencies of the four trees at subfamily level. That is, the position represented by a red dotted lines as shown in Figure S1. [file 12862_2020_1623_MOESM5_ESM.jpg]
